# Centenarians and supercentenarians: a black swan. Emerging social, medical and surgical problems

**DOI:** 10.1186/1471-2482-12-S1-S36

**Published:** 2012-11-15

**Authors:** Marco Vacante, Velia D’Agata, Massimo Motta, Giulia Malaguarnera, Antonio Biondi, Francesco Basile, Michele Malaguarnera, Caterina Gagliano, Filippo Drago, Salvatore Salamone

**Affiliations:** 1 Department of Senescence, Urological and Neurological Sciences, Cannizzaro Hospital Via Messina 829, 95125, University of Catania, Italy; 2 Department of Biomedical Sciences, Via S. Sofia, 87, 95123, University of Catania, Italy; 3 International PhD programme in Neurosciences, University of Catania, Italy; 4 Department of General Surgery, Section of General Surgery and Oncology, Vittorio Emanuele Hospital, Via Plebiscito 628 University of Catania, 95123 Catania, Italy; 5 Department of Ophtalmology, Via S. Sofia 78, 95123, University of Catania, Italy

## Abstract

The Black Swan Theory was described by Nassim Nicholas Taleb in his book “The Black Swan”. This theory refers to “high-impact, hard-to-predict, and rare events beyond the realm of normal expectations”. According to Taleb’s criteria, a Black Swan Event is a surprise, it has a major impact and after the fact, the event is rationalized by hindsight, as if it had been expected. For most of human history centenarians were a rare and unpredictable phenomenon. The improvements of the social-environmental conditions, of medical care, and the quality of life caused a general improvement of the health status of the population and a consequent reduction of the overall morbidity and mortality, resulting in an overall increase of life expectancy. The study of centenarians and supercentenarians had the objective to consider this black swan and to evaluate the health, welfare, social and economic consequences of this phenomenon.

## Introduction

The term “Black Swan” comes from the 17th century European belief that the existence of a black swan was impossible. After a hundred years black swans were discovered in Western Australia and so the term started to indicate that a perceived impossibility may come to pass [1]. The Black Swan Theory refers to high-impact, hard-to-predict, and rare events beyond the realm of normal expectations. The theory was described by Nassim Nicholas Taleb in his 2007 book “The Black Swan”. Taleb regards almost all major scientific discoveries, historical events, and artistic accomplishments as "black swans" — undirected and unpredicted [1]. Healthy centenarians are a living example of successful aging free from chronic diseases causing permanent injuries and from reduced mental and physical functions [2]. For most of human history centenarians were a rare and unpredictable phenomenon [3]. Although numerous studies have characterized the centenarian phenotype according to metabolic, endocrine, immune, physical and cognitive functions, little work has emerged that describes the health histories associated with exceptional longevity [4-7]. The improvements of the social-environmental conditions, of medical care, and the quality of life caused a general improvement of the health status of the population and a consequent reduction of the overall morbidity and mortality, resulting in an overall increase of life expectancy. Around the 1970s, the progressive decline of mortality (1–2% per year) in individuals over 80 years old has increased in all industrialized countries, so that the number of centenarians has augmented about 20-fold [8]. The term “supercentenarians” describes the subjects who had reached 110 years of age, in a validated manner. Their prevalence is estimated to be 0.3-0.5% of the centenarians [9]. Supercentenarians should be considered as exceptional individuals having a particularly efficient network, able for exceptional performances to slow down the numerous pathological conditions determining the aging process, and stimulating the factors resulting in resistance against diseases, increasing this way the survival [10].

### Factors that influence the longevity

Many studies suggested that some factors are important to longevity in centenarians: 1) heredity, role of specific genes and family history [11] 2) general health and lifestyle, i.e. weight, diet, amount of physical exercise, smoking habits [12,13] 3) education level [14] 4) personality [15,16]. The largest population of centenarians are widowed women [17]. The Okinawa Centenarians Study (OCS) has shown several different factors that have contributed to the large number of centenarians there. These factors are: 1) a diet based mainly on grains, fish and vegetables instead of meat, eggs, and dairy products; 2) low-stress lifestyles, compared to the mainland inhabitants of Japan; 3) caring community and active work until an older age than the average age in other countries; 5) a strong role of spirituality, with involvement in spiritual matters and prayer that ease the mind of stress and problems [18-20]. Human longevity is due to genetics, age, sex, ethnicity and environment of the study population. [21-23]. Whether prolonged caloric restriction (CR) increases average or maximum lifespan or promotes a more youthful physiology in humans at advanced ages is not yet known. However, available epidemiological evidence indicates that CR may already have contributed to an extension of average and maximum life span in older Okinawans and appears to have lowered risk for age associated chronic diseases in other human populations [24].

### General conditions of centenarians

Centenarians display extremely variable clinical conditions. On the one hand, there are frail individuals among them with polypathologies, being an expression of the terminal deterioration related to the progressive increase of the medium life span. On the other hand, there are subjects without particular disease conditions or cognitive disorders. Some of them present, however, signs of the advanced aging process, such as hypoacusia, visual disorders, limited locomotor capacities, etc. [25,26]. A consortium of 20 university departments of geriatrics and gerontology conducted the Italian Multicentric Study on Centenarians (IMUSCE), in order to assess the socio-economic, clinical and biological conditions of Italian centenarians. According to the IMUSCE criteria, based on psychophysical status and autonomy, centenarians can be classified in three groups as follows: Group A: centenarians in good health status; Group B: centenarians in an intermediate health status. Group C: centenarians in bad health status. Group A represented 20.0% of the total pool, Group B amounted to 33.4%, and the Group C was 46.6%. The centenarians of Group A presented normal activities of daily living (ADL) values, and 47.9% of them were autosufficient in all functions; 5.7% of them were independent in all instrumental activities of daily living (IADL) items. These data confirm that the centenarians of Group A are free of invalidating chronic diseases, are autonomous, maintain good physical and cognitive capacities, however, have not maintained any social or productive activities. Therefore, they cannot be considered as prototypes of successful aging [26].

### Supercentenarians

The supercentenarians display an elevated percentual occurrence of alterations which do not have a deterministic role in the survival: (cataract, osteoporosis, bone fractures, etc.), and a low prevalence of more significant, chronic degenerative pathologies. Generally they reach 100 years of age in good health, and only after 105 years of age start to manifest age-dependent alterations with high variability [9]. Usually their death cause is not correlated to the typical pathologies of aged people, such as cancer, stroke, myocardial infarction, etc. [22,27]. The decreased prevalence of various pathologies widely documented in the centenarians, as compared to the elderly, seems to be present also in the supercentenarians, compared to the centenarians. Even the dementia of various clinical aspects, which is the only disease condition more frequent in the elderly than in the centenarians [28] seems to be of lower prevalence in the supercentenarians, as compared to the centenarians [9].

### Immunity and centenarians

Ageing of the immune system relates to both innate and adaptive immunity although the innate one is regarded to be better preserved than adaptive one [29-31]. The age-related alterations of adaptive immunity include a decline of naive T lymphocytes and accumulation of memory/effector T cells, reduction in the number of B cells, up-regulation of the inflammatory responses and dysregulation of the Th1/Th2 system. The increased number of Natural Killer (NK) cells with well-preserved cytotoxic function is a characteristic feature of the innate part of ageing immune system [32]. The process of successful ageing, (i.e. ageing in good psychophysical conditions) [33] is immunologically characterized by preserved lymphoproliferative responses and NK cytotoxic activity as well as conserved antigen presentation [34]. Healthy centenarians show normal number of T lymphocytes, increased production of immunoglobulins, lack of organ-specific autoantibodies, well preserved NK activity and retained proliferative capability of T lymphocytes [35]. The remodelling of the immune system occurring with age is capable of creating a hostile environment for the growth of cancer cells in centenarians [36]. In fact an age-dependent increase of CD8+ CD28 – T cells having a high cytotoxic capacity, both in percentage and absolute number, has been found in elderly people and centenarians [37,38]. Moreover, an increased number and percentage of NK cells (CD16+, CD56+, CD57+) has been found in centenarians with the highest NK function and number of NK cells have preserved endocrine conditions and muscle mass [39,40]. Thus the increase with the age of cells with NK features could cause a prevailing of the innate immunity in the oldest old, which might create an unfavourable environment for neoplastic growth [41-44].

### Hormonal status in centenarians

It is well known that physiological changes in the neuroendocrine system may be related to the process of aging. A study revealed several differences in the neuroendocrine and metabolic status of centenarians, compared with other age groups, including the lowest serum concentrations of leptin, insulin and T3, and the highest values for prolactin. LH and FSH levels were comparable with those in the elderly and postmenopausal groups, but they were significantly higher than in younger subjects. GH concentrations in centenarians were lower than in younger women. It has been also demonstrated that BMI in centenarian subjects does not differ significantly from BMI of younger subjects but it is lower than that found in early elderly subjects [45]. Several studies in humans have shown that longevity is associated with a significant improvement in glucose handling - mainly, a rise in insulin sensitivity and a decline in plasma insulin-like growth factor I levels [46]. Moreover Paolisso et al. demonstrated that centenarians compared with aged subjects had a preserved glucose tolerance and insulin action despite to the decline in insulin action due to advancing age [47].As regards thyroid activity, contrasting results have been revealed. In fact it has been showed an age-related decline of the TSH levels and a significant increase of the reverse T_3_ (rT_3_) concentrations in centenarians by comparison to old controls. These findings may be related to an age-dependent reduction of the 5'-deiodinase activity [48]. Other studies showed that the distribution of serum TSH shifts progressively to higher concentrations with age, appearing to be a continuum that extends even to people with exceptional longevity. The inverse correlation between TSH and FT4 in this study populations suggests that changes in negative feedback may contribute to exceptional longevity [49].

### Energy, carnitine and antioxidants

L-carnitine and its short-chain esters facilitate long-chain fatty acid transport across the inner mitochondrial membrane for β-oxidation. It thereby promotes energy availability and prevents toxic accumulation of long-chain fatty acids [50,51]. Acetyl-L-carnitine (ALC) is an ester of L-carnitine, a trimethylated aminoacid; it is synthesized in the central nervous system (CNS), liver, and kidney via the action of ALC transferase and stored in skeletal muscle, both in the free form and as an ester with acetyl groups [52]. ALC concentration is age and gender dependent. ALC controls the transport of long-chain fatty acids in mitochondria and then their β-oxidation, and it ensures the minimum necessary level of acetyl-coenzyme A required for energetic cellular metabolism [53]. ALC is the most abundant L-carnitine ester in the nervous system, being fundamental for lipid metabolism and polyunsaturated fatty acids synthesis in neuronal membrane. It is also able to improve neuronal metabolism by increasing the use of glucose and eliminating oxidative metabolites and to enhance the activities of electron transport chain enzymes [54-56]. Caloric restriction, low levels of oxidative stress and changes in glucose handling seem to be the most compelling factors relating longevity and metabolism. Caloric restriction has been shown to reduce body temperature, improve insulin sensitivity (by lowering fasting plasma glucose and insulin levels and improving insulin action), lower fasting plasma free insulin-like growth factor I (IGF-I) and dehydroepiandrosterone (DHEA) levels and improve thyroid activity. Together, these actions result in two main effects: lowering energy production in the mitochondrial complex and minimizing DNA damage and thus genomic instability. With regard to energy production in the mitochondrial complex, an ad libitum diet is associated with enhanced production of reactive oxygen substances (ROSs) that activate several cascade mechanisms resulting in impairment of enzyme activity. In contrast, caloric restriction lowers mitochondrial activity, which decreases the production of ROSs, leading to a secondary improvement in intracellular metabolism. Cellular aging is slowed by decreasing the production of ROSs, which leads to reduced DNA damage [57]. The principal target of ALC’s action is mitochondrial DNA, on which it exerts an antioxidant effect and stimulates mitochondrial DNA synthesis [58]. Moreover ALC supplementation may reduced significantly both physical and mental fatigue and improved physical activity and cognitive status [59]. Various mechanism can explain the therapeutic effect of ALC, such as the beneficial effects of ALC on mitochondrial alterations and on the progressive impairment of neurotransmission, the correction on deficits of cellular energy supply. Carnitine and its derivative, ALC affect other cellular functions, including maintenance of key proteins and lipids of the mitochondria at sufficient levels, proper membrane orientation and maximum energy production [60].

### Cardiovascular risk factors and oxidative stress in centenarians

Several studies have shown that centenarians have better cardiovascular risk profiles compared to younger old people. Some reports have revealed that cardiovascular diseases (i.e. hypertension, diabetes, angina and/or myocardial infarction) are less common in centenarians respect to 70 and 80 years old persons [61]. Centenarians have high levels of the natural antioxidants vitamins A and E [62] that may be protective from atherosclerosis. But they present enhanced coagulation enzyme activity, elevated plasma fibrinogen and homocysteine levels, which represent thrombotic risk factors for middle-aged individuals [63-66]. Moreover centenarians present elevated levels of IL-18, which is a proinflammatory cytokine that appears to be involved in atherosclerosis [67]. Gangemi et al demonstrated the apparent paradox of elevated serum IL-18 with no vascular signs in centenarians can be explained by the presence of high IL-18 binding protein levels (a protein that neutralizes the activity of IL-18) in these subjects [68]. A study showed that indices of oxidative stress (reaction products of malondialdehyde with thiobarbituric acid (TBARS) and lipid hydroperoxides (LPO), were lower in centenarians than in aged subjects. In contrast, reduced/oxidized glutathione ratio (GSH/GSSG) and plasma concentrations of antioxidant defenses (plasma vitamin E and C) were more elevated in centenarians than in aged subjects [69]. A study by Rabini et al showed that there is an age-associated trend in the platelet membrane concentrations of a biomarker of oxidative stress (MDA), but centenarians showed platelet membrane concentrations of MDA lower than elderly subjects and similar to the levels found in an adult group. These difference is associated with a decreased platelet activation and therefore might exert a protective role against cardiovascular accidents, as platelet activation is a key event in the initiation and progression of arteriosclerosis [70]. As regards the lipids analysis in centenarian populations, contrasting results have been revealed. There are findings about decreased or increased values of total cholesterol, LDL- and HDL-cholesterol, triglycerides, ApoB100, and ApoA1. As regards the serum levels of lipoprotein(a), increased or unchanged values have been observed [71-73] Total cholesterol decreased with advancing age, but high-density lipoprotein maintained at high levels, consistent with a previously reported lipoprotein phenotype of exceptional longevity in centenarians [74]. Receputo et al. observed noted that total cholesterol values in a group of male centenarians were within the normal range and were significantly lower than in elderly male subjects [75]. These data support the protective role of low total cholesterol values also with regard to longevity [76]. Even if blood pressure progressively increases with age [77], Gareri et al. have not found it in centenarians [78]. The lesser frequency of hypertension in centenarians certainly depends on genetic and constitutional factors, but also their style of life and alimentary habits (decreased sodium intake, increase of calcium and potassium intake) could be determinant [79,80]. The extraordinary longevity of centenarians can not be explained even if hypertension, that is a risk factor for cardiovascular and cerebrovascular morbidity and mortality, is present for all the ages of life [78].

### Cancer and longevity

Centenarians appear to “outlive” the risks for many of those conditions that are common causes of death for those who die in their 70s, 80s and 90s, such as cancer and myocardial infarction [81]. The analysis of demographic data indicates that cancer incidence and mortality show a levelling off around the age of 85-90 years, and suggests that oldest old people and centenarians are protected from cancer onset and progression [82]. Accordingly, studies on autopsy records revealed that long-lived people and centenarians are characterised by a lower than expected incidence, metastatic rate and mortality for cancer [83,84]. Moreover an autoptic study performed on 140 centenarians, has revealed the presence of various types of cancer in 16.3%, as compared to the average elderly showing 39.0%, and there was a lower aggressivity of metastases, too (24.0% vs. 55.0 %) [85]. The complex relationship between the change of apoptosis susceptibility with age and cancer prevalence in the oldest old can predict that gene products and polymorphisms of genes are involved in apoptosis [86]. A particular attention deserves the relationship between apoptosis, its age-related changes, and cancer susceptibility in the oldest old [87]. ApoJ is a gene which appears to play an important role in aging, apoptosis and cancer but whose role is still unclear [88]. In cancer cells, this gene confers cytoprotection by inhibiting apoptosis, although there are reports indicating a pro-apoptotic function of ApoJ in other tumor-derived cell lines [89] In recent years the extensive genotypisation of centenarians led to study polymorphisms which are claimed to be able to affect individual’s cancer susceptibility, namely those located in the anti-oncogene p53, in the oncogene HRAS1, in asset of genes involved in carcinogens metabolism, i.e. cytochrome P450 oxidases (CYP) and glutathione transferases (GST), in a gene whose mutations are risk factor for breast cancer (BRCA1) and in a gene whose activity is a potential risk factor for prostate cancer (SRD5A2) [41] Nevertheless it is difficult to interpret the net difference in the prevalence of cancer in extreme longevity: in the Okinawa Centenarian Study (OCS) [22], cancer is absent, while in the New England Centenarian Study (NECS) [27], it is present in 25% of the supercentenarians, however, all of them were previously treated, and none of them were active. This is a condition which justifies the presence of cancer in the supercentenarians. Some of centenarians had been affected by cancer in their life, but they survived, even though during an historical period when cancer treatment was not as developed as in the present days [90,91]. Thus is reasonable to conclude that centenarians are people endowed with a peculiar resistance to cancer [41] The relationship between polymorphism associated with cancer susceptibility and human longevity is complex and the studies so far performed provided insights on some mechanisms involved in human longevity. At the moment the role of the immunosenescence on cancer incidence is an extremely debated argument [92]. It has been suggested that the immunosenescence is not an inevitable and progressive decline of all immune functions, but rather the result of a continuous remodelling process in which several functions are reduced, others increased, while others remain unchanged [93]. Studies of the immune system of centenarians, spotlighted that one of the main factors of longevity may be represented by well functioning immune system which allows the prevention of the main age-related pathologies including cancer, as death from cancer may decline at very old age. In elderly subjects practicing regularly moderate exercise training, some aspects of immunosenescence are attenuated or improved, both innate immunity and acquired immunity, as the reduction of memory cells and the increase of naïve T cells [94,95] increase further the production of primary antibody response [96].

## Conclusion

Life expectancy has dramatically increased over the last few centuries of human history and it continues to increase. Before about 2000 BC, the number of births per year was under 1 million; until roughly 1000 AD annual global births ran at less than 10 million; only since 1970 have more than 100 million babies been added to the human population each year [97]. If the chance of surviving to age 100 is about 1 in 20 million when life expectancy is 20 and about 1 in 80,000 when life expectancy is 40 (a level not reached in Western Europe until the early 19th century), then centenarians must have been exceedingly rare in most countries before the modern era. The identification of a Black Swan Event, basing on Taleb’s criteria, includes that the event is a surprise, the event has a major impact and after the fact, the event is rationalized by hindsight, as if it had been expected [1]. The Black Swan event is isolated and unpredictable. One naturally tends to give a retrospective justification of his appearance to make it less random than it actually is. Unless there is some secret of longevity that has enabled some humans to transcend the death rates that governed the fate of nearly all their contemporaries, most accounts of centenarians in earlier centuries must be inaccurate [98]. In Italy the number of centenarians has risen from 49 in 1921 to 1304 in 1981, to 1660 in 1990, to 4000 in December 1995 [99,100]. The growth in the number of centenarians has garnered significant attention over the past 20 or so years. One study reported Medicare data indicating that, in 2000, there were 32,920 centenarians and that, of these, 0.3% were age 110 and older [101]. Furthermore it is estimated that seven in 1,000 people born at the turn of the last century lived to become centenarians and that one in 100,000 lived to be 110 or older [102]. A better understanding of the biochemical and neuroendocrine determinant of aging has put interventions on a somewhat more solid scientific foundation to combat a few of the deleterious consequences of aging. Many studies are concerned with elucidating the regulatory mechanisms of aging at the cellular level and we expect that they will also have practical implications that translate into real therapeutic interventions in the coming decades [103]. The centenarians can still be heterogeneous enough to warrant careful phenotyping for the purposes of discovering different potential genetic and environmental correlates of exceptional longevity. In addition, there may be distinctive genetic and environmental interactions involved in the exceptional longevity of men versus women. According to the U.S. Department of Census, the number of centenarians could cross the 4 million mark by 2050 [104] In the US, the rise in health care expenditures associated with the rapid increase in the elderly population will likely place additional pressures on the Medicaid and Medicare programs, as well as private insurers, to control health care costs. Efforts to control costs will likely have a negative impact on both the supply of and demand for health workers. Nevertheless, the growth of centenarians and even more of supercentenarians, deserves a detailed evaluation as regards the enormous social, economic and health impacts. The high impact of longer lifespans has consisted in the last years in a gradual alteration of the way individuals want to spend their time during lives, thus leading to drastic revision of education, health, employment, retirement and other policies. The study of centenarians and supercentenarians had the objective to consider this black swan to better understand the health, welfare, social and economic consequences of this phenomenon.

## Competing interests

The authors declare that they have no competing interests.

## Authors' contributions

MV, MM: conception and design, drafting the manuscript, given final approval of the version to be published; VDG, MM, GM, CG, FD: drafting the manuscript, given final approval of the version to be published; FD, SS, AB, FB: critical revision, given final approval of the version to be published.
